# Investigation of the RbCa molecule: Experiment and theory

**DOI:** 10.1016/j.jms.2015.01.006

**Published:** 2015-04

**Authors:** Johann V. Pototschnig, Günter Krois, Florian Lackner, Wolfgang E. Ernst

**Affiliations:** Institute of Experimental Physics, Graz University of Technology, Petersgasse 16, A-8010 Graz, Austria

**Keywords:** Helium nanodroplets, Spectroscopy, Alkali–alkaline earth molecules, Excited states, Rubidium, Calcium, Ab initio

## Abstract

We present a thorough theoretical and experimental study of the electronic structure of RbCa. The mixed alkali–alkaline earth molecule RbCa was formed on superfluid helium nanodroplets. Excited states of the molecule in the range of 13 000–23 000 cm^−1^ were recorded by resonance enhanced multi-photon ionization time-of-flight spectroscopy. The experiment is accompanied by high level *ab initio* calculations of ground and excited state properties, utilizing a multireference configuration interaction method based on multiconfigurational self consistent field calculations. With this approach the potential energy curves and permanent electric dipole moments of 24 electronic states were calculated. In addition we computed the transition dipole moments for transitions from the ground into excited states. The combination of experiment and theory allowed the assignment of features in the recorded spectrum to the excited $3{}^{2}\Sigma^{+}$, $4{}^{2}\Sigma^{+}$, $3{}^{2}\Pi$, $5{}^{2}\Sigma^{+}$, $4{}^{2}\Pi$, $6{}^{2}\Sigma^{+}$, $6{}^{2}\Pi$, and $7{}^{2}\Pi$ states, where the experiment allowed to benchmark the calculation. This is the first experimental work giving insight into the previously unknown RbCa molecule, which offers great prospects in ultracold molecular physics due to its magnetic and electronic dipole moment in the ${}^{2}\Sigma^{+}$ ground state.

## Highlights

• Helium nanodroplet spectroscopy was used to investigate excited states of RbCa.
• The spectral range from 13 000 to 23 000 cm^−1^ was covered by multi photon ionization.
• Ab initio calculations were performed for 24 electronically excited states of RbCa.
• The spectral features were assigned to the calculated potentials.

## Graphical abstract

**Figure:**
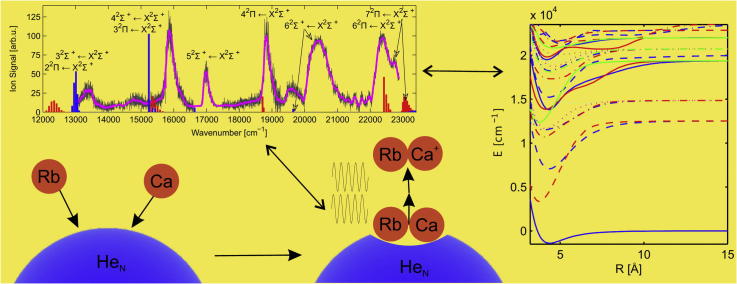

## 1 Introduction

Alkali–alkaline earth (Ak–Ake) molecules, such as RbCa, are of growing interest in ultracold molecular physics, because they possess a permanent electric dipole moment in combination with a magnetic dipole moment in their ${}^{2}\Sigma^{+}$ ground state. These properties enable new exciting applications and experiments, such as precise measurements of fundamental physical constants [1,2] or simulations of spin models in optical lattices [3,4]. The replacement of the Ake by Yb leads to a class of molecules with similar features which have been studied theoretically [5–10] as well as experimentally in combined traps of ultracold Ak and Yb atoms [11–14]. Ake monohalides, such as CaF or SrF [15–18], also possess a magnetic and permanent electric dipole moment in their ${}^{2}\Sigma^{+}$ ground state. While Ake monohalides are typically produced at elevated temperatures and need to be cooled as molecules, Ak and Ake atoms are individually cooled into the ultracold temperature regime for a subsequent laser assisted formation of molecules. The latter method has been successfully applied to form ultracold homo- and heteronuclear Ak diatomic molecules [19,20] and does not require the more difficult cooling of the many degrees of freedom in molecules.

Due to this interest in Ak–Ake molecules their ground states have been studied extensively by theory [21–28]. Experimentally, LiBa and LiMg were investigated by means of spectroscopy [29–32] also in combination with calculations [33]. LiCa is the best studied Ak–Ake molecule including several experiments [34–37] and theoretical calculations [38,34,37,39]. Among the Ak–Ake molecules, RbSr appears to be the most promising candidate for the preparation of ultracold ground state molecules. Rb and Sr atoms have been prepared in combined traps [40,41] and quantum degenerate mixtures have been obtained [40]. Consequently, excited states of RbSr were investigated theoretically [42–44] and experimentally on He nanodroplets [45,46].

RbCa represents the other interesting molecule, because Rb and Ca are well under control in ultracold atomic physics. Ultracold Rb_2_ ground state molecules [20] as well as Bose Einstein condensates of Rb [47] and Ca [48] have been reported. Furthermore, a combined trap of Rb atoms and Ca^+^ cations has been applied in the study of cold reactive collisions [49]. The electronic structure of RbCa is unknown; to the best of our knowledge neither calculations nor experimental investigations of excited states have been reported. The ground state has been addressed in recent calculations [25] as well as the ground and excited states of the RbCa^+^ cation [49–51]. Considering these prospects for RbCa, a spectroscopic investigation of this molecule is of great interest. Gas phase formation of RbCa is impeded by the very different vapor pressure curves of Rb and Ca. Here, matrix isolation spectroscopy comes to mind, which has been the starting point of many free radical studies in the past. Many examples were reported in a special issue in 2000 [52] and we want to draw special attention to the extensive and pioneering work by Marilyn Jacox [53]. While perfect for the preparation of radicals in the cold environment of a rare gas host, matrices usually induce some perturbation and non-negligible spectral line shifts. About 20 years ago, doping helium nanodroplets with foreign atoms and molecules [54,55] turned out to evolve as a new form of matrix isolation spectroscopy, named helium nanodroplet isolation (HENDI) spectroscopy [56]. As the new nano-matrix is created in a molecular beam apparatus, most methods of molecular beam spectroscopy, including double-resonance techniques, can be applied. A detailed survey is given in a recent book chapter [57], which also addresses the much smaller influence on spectral lines in comparison with conventional matrix isolation spectroscopy. Helium droplets have a constant temperature of 0.37 K for ^4^He [57,58] maintained by evaporative cooling, which greatly simplifies excitation spectra because the dopant molecules are in their vibronic ground state. Ak and Ake atoms have been previously investigated with HENDI spectroscopy [59–62] and except for Mg, were all found to reside on the droplet surface. Besides atoms, several homo- and heteronuclear diatomic molecules consisting of alkali atoms [63,64] as well as Ak trimers [65–67] have been studied on He nanodroplets. Recently HENDI spectroscopy was successfully extended to Ak–Ake diatomic molecules [37,45,46], which were formed on helium droplets by applying a sequential pick up scheme.

In this manuscript we present an application of the HENDI approach to the RbCa molecule. The experimental results for the excited states have been obtained by resonance enhanced multi-photon ionization time-of-flight (REMPI-TOF) spectroscopy. In our theoretical treatment the potential energy curves (PECs), the transition dipole moments (TDMs) and the permanent electric dipole moments (PEDMs) were determined for the neutral RbCa molecule with post Hartree–Fock molecular orbital theory.^2^ The combination of experiment and calculations enables the assignment of experimentally recorded features and allows for an estimate of the accuracy of the calculation. The knowledge gained from this work is essential for experiments envisaging the creation of ultracold molecules from quantum degenerated gas mixtures, which is based on the navigation via various potential energy curves with multiple lasers to the molecular ground state [2,68,69].

## 2 Theory

We calculated the ground state and the excited states of the diatomic molecule RbCa with post Hartree–Fock molecular orbital theory applying the MOLPRO software package [70]. Potential energy curves and electronic properties of the excited states were computed by a multireference configuration interaction [71] calculation of second order based on a multiconfigurational self consistent field calculation [72]. The inner core of the Rb atom, containing 28 electrons, was described by the relativistic effective core potential ECP28MDF [73] and the corresponding basis set. The basis set was slightly modified and a core polarization potential was applied as described in Ref. [42]. The 10 innermost electrons of the Ca atom were replaced by the effective core potential ECP10MDF [74]. The cc-pV5Z basis set [75] was applied with a core polarization potential following the approach in Ref. [37]. The calculation was performed in the C_2*v*_ point group, the orbitals will be given in the program-specific order of the irreducible representations (A_1_/B_1_/B_2_/A_2_). The active space comprised 19 electrons in 37 orbitals (17/9/9/2). Out of them, eight orbitals (4/2/2/0) were kept doubly occupied at all times, but were included in the coefficient optimization. In the multiconfigurational self consistent field calculation (15/9/9/5) states were determined with doublet symmetry and (3/2/2/1) states for the quartet symmetry. This was necessary in order to obtain reasonable results for the 37 states calculated with the multireference configuration interaction, including (12/7/7/3) doublet states and (3/2/2/1) quartet states. The states, determined in the irreducible representations of C_2*v*_ for computational ease, can be assigned to diatomic states by inclusion of additional information, e.g. based on their energetic order. This approach yielded a good agreement of the results with atomic energy levels in the asymptotic limit at 30 Å, as is shown in Table 1. The calculated values agree within 3.5% with the values reported in the NIST-database [76]. The excitation energies of diatomic states corresponding to atomic D-states are slightly overestimated. For all other states an underestimation of the energies is observed.

TDMs between the ground and excited states were computed by the same level of theory. At 30 Å, the majority of excited states have a TDM of zero in agreement with the selection rules for the corresponding atomic transitions. For the $8{}^{2}\Sigma^{+}$ and $6{}^{2}\Pi$ states a TDM^2^ of 8.96 ${\text{e}}^{2}{\text{a}}_{0}^{2}$ was obtained at 30 Å, corresponding to a line strength of *S*_ik_/*g*_k_ = 8.13 ${\text{e}}^{2}{\text{a}}_{0}^{2}$ for the Ca (4s4p ^1^P°) ← Ca (4s^2^ ^1^S) transition as found in the NIST-database [76]. The literature value for the Rb (5p ^2^P°) ← Rb (5s ^2^S) transition is *S*_ik_/*g*_k_ = 8.94 ${\text{e}}^{2}{\text{a}}_{0}^{2}$ [76], the corresponding $2{}^{2}\Sigma^{+}$ and $1{}^{2}\Pi$ states show a TDM^2^ of 10.12 ${\text{e}}^{2}{\text{a}}_{0}^{2}$. The $9{}^{2}\Sigma^{+}$ and $7{}^{2}\Pi$ states also have a noticeable TDM^2^ of 0.10 ${\text{e}}^{2}{\text{a}}_{0}^{2}$ at 30 Å. For the corresponding Rb (6p ^2^P°) ← Rb (5s ^2^S) transition a value of *S*_ik_/*g*_k_ = 0.06 ${\text{e}}^{2}{\text{a}}_{0}^{2}$ [76] is given.

The ground state of the cation RbCa^+^ was investigated with the same basis set and methods. An ionization potential of IP = 33482.18 cm^−1^ was determined at 30 Å, comparable to the Rb atom with an ionization potential of IP = 33690.81 cm^−1^ as found in the NIST-database [76].

## 3 Experiment

The following paragraph provides a short overview of the experimental setup. Details can be found in Refs. [65,77,78]. A beam of He nanodroplets is generated by a supersonic jet expansion of He gas at 60 bar through a 5 μm nozzle at 15 K. The obtained droplet size distribution shows a maximum at ${\overset{ˆ}{N}}_{60\text{,}15}=6000$ and a mean value of ${\overset{¯}{N}}_{60\text{,}15}=14\;000$, corresponding to radii of ${\overset{ˆ}{R}}_{60\text{,}15}=40$ Å and ${\overset{¯}{R}}_{60\text{,}15}=54$ Å, respectively, assuming spherical droplets [58].

The established He cluster beam is extracted by a 5 μm skimmer and subsequently enters the pickup chamber. There it passes through two resistively heated pickup cells, which contain the dopant materials Rb and Ca. The probability of a dopant pickup by the He droplets follows a Poisson distribution and depends on the length of the pickup cell, the droplet size and the vapor pressure of the dopants in the pickup cells [79]. The molecular beam and pickup conditions for different experiments may vary and the signals were optimized for the respective conditions. The optimum pickup temperature to obtain a maximum RbCa signal has been found to be around $T_{\mathrm{Rb}}=85$ °C and $T_{\mathrm{Ca}}=400$ °C.

An excitation spectrum for RbCa molecules on He nanodroplets was recorded over a large wavelength range with resonance enhanced multi-photon ionization time-of-flight (REMPI-TOF) spectroscopy, using a dye laser (Lambda Physik FL 3002) to excite the molecule and a fraction of the pump laser (Radiant Dyes RD-EXC 200 XeCl laser, 26 ns pulse duration, 100 Hz) to ionize it. Above an energy of ∼14X 000 cm^−1^ only the dye laser was used for excitation and ionization. A time-of-flight mass spectrometer (Jordan D-850 AREF) with angular reflectron served to record the ion signal. Laser induced fluorescence spectroscopy was applied for an investigation of molecular transitions found in the REMPI-TOF spectrum, however no fluorescence light originating unambiguously from excited RbCa was observed in the experiment.

## 4 Results and discussion

### 4.1 Overview

**Fig. 1:**
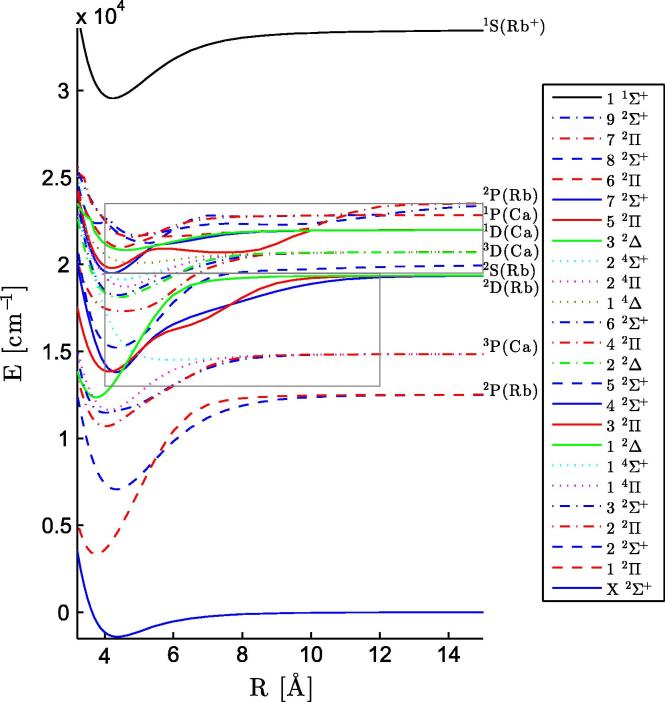
PECs of electronic states of RbCa and the ground state of RbCa^+^ are displayed, for each multiplicity and symmetry a distinct color is used. Note, that a few PECs show the same color and line style. At the long range limit the corresponding atomic energy levels are denoted. All states above the $9{}^{2}\Sigma^{+}$ state have been neglected, except for the $1{}^{1}\Sigma^{+}$ state of the molecular ion. The gray rectangles mark the areas that are shown in more detail in Figs. 5 and 9. (For interpretation of the references to color in this figure legend, the reader is referred to the web version of this article.)

In total we calculated 24 electronic states of the neutral RbCa molecule, corresponding asymptotically to the ground states of the atomic constituents, four excited states of Rb, and four excited states of Ca. The potential parameters of the 24 states have been determined and collected in Table 2, as well as the values for the ground state of the RbCa^+^ cation. There are two strongly bound states with an equilibrium radius smaller than 4 Å, the $1{}^{2}\Pi$ and $1{}^{2}\Delta$ states. These strongly bound states as well as a very weakly bound $1{}^{4}\Sigma^{+}$ state were also observed for LiCa [37] and RbSr [42]. The PECs of the electronic states are displayed in Fig. 1. Avoided crossings appear where two PECs of the same symmetry approach each other, altering the PECs and electronic properties of the involved states. Within the energy range of Fig. 1, two zones of prominent avoided crossings are marked by rectangles. The corresponding portions have been blown up and are depicted together with the plots of TDMs and PEDMs in Figs. 5 and 9. There, strong changes in the TDMs and PEDMs are related to the avoided crossings.

An excitation spectrum of RbCa has been recorded with REMPI-TOF spectroscopy in a spectral range of 13 000–23 000 cm^−1^. The experimental spectrum has been divided in three parts, which will be shown and addressed individually in the following sections. The energetic positions of the rising edges of the recorded spectral features are given in Table 2. Note that for transitions which show a steep rising edge followed by an extended blue tail (i.e. the potential minima of the ground and excited state have a similar equilibrium separation), the rising edge can be assumed to coincide with the potential minimum, because all molecules are initially in their vibronic ground state [37,45,46]. For other transitions, the position of the rising edge serves as an upper limit for the potential minimum. The spectra are comprised of peaks recorded with various laser dyes. They have been offset-corrected, but not scaled, with laser pulse energies within a range of 0.5–1.5 mJ, depending on the laser dye. Therefore, the relative heights of the peaks shown in the figures can only be tentatively compared to each other.

It can be expected that the line shift introduced by the helium environment is small for RbCa. Rb and Ca are both heliophobic atoms, like all alkali atoms and most alkaline earth atoms. They reside on the surface of the helium droplet, which limits the perturbations of the electronic states to some extent. Recently, a REMPI-TOF spectrum of LiCa formed on helium nanodroplets was compared with gas phase measurements [37]. The results show a small line shift of a few cm^−1^ in the REMPI-TOF spectrum in agreement with a surface location. Additionally, a broad (10–100 cm^−1^) phonon wing accompanies each vibronic transition resulting in an unresolvable merged structure for most transitions.

For a more thorough comparison of the theoretical and experimental data, the Franck–Condon factors (FCFs) have been used. The FCFs for the PECs in Fig. 1 were calculated using the program Level 8.0 of Le Roy [80]. The corresponding transition probabilities (FCF∗TDM^2^) were determined by multiplying the FCFs with the square of the TDMs at 4.4 Å (ground state minimum) and are indicated in Figs. 4, 6, and 8 as vertical bars. The calculations allowed to assign the peaks in the experimental spectra. Generally, the theoretical values for the molecular transitions lie slightly lower in energy than the experimental values (∼40–400 cm^−1^) for all states below an excitation energy of 22 000 cm^−1^.

### 4.2 Ground state

**Fig. 2:**
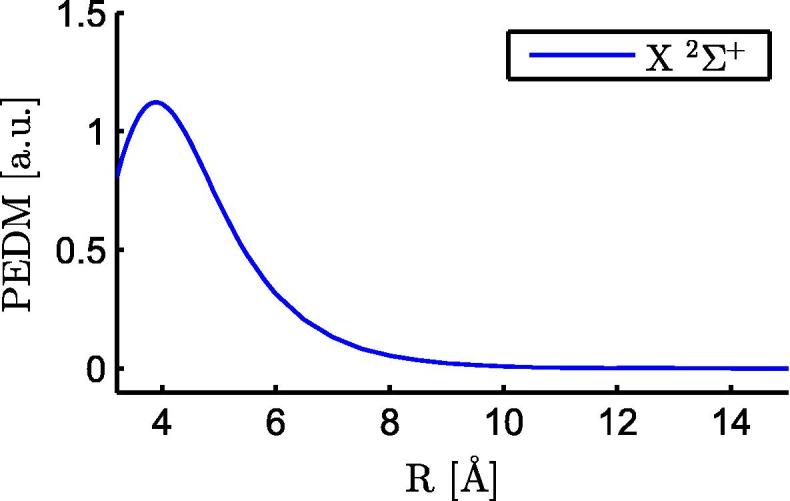
This figure displays the PEDM of the ground state of RbCa as a function of the internuclear separation of the atoms.

The results for the ground state can be compared to previous calculations for RbCa as well as experimental and theoretical results for similar molecules. The parameters determined for the ground state potential (*D*_*e*_ = 1406 cm^−1^, *r*_*e*_ = 4.37 Å, $\omega_{e}$ = 58 cm^−1^) lie in between the experimental values for LiCa (*D*_*e*_ =  2605 cm^−1^, *r*_*e*_ = 3.36 Å, $\omega_{e}$ = 202 cm^−1^ [36]) and the theoretical values for RbSr (*D*_*e*_ = 1041/916/1273 cm^−1^, *r*_*e*_ = 4.67/4.72/4.59 Å, $\omega_{e}$ = 38/36/42 cm^−1^ [44,25,42]). These intermediate values for RbCa are reasonable because both RbSr and LiCa share one atom with RbCa, paired with a heavier (Sr) or lighter (Li) atomic partner. In a previous calculation for ground states of several Ak–Ake diatomic molecules Gopakumar et al. [25] found for RbCa a potential depth of *D*_*e*_ = 921 cm^−1^, an equilibrium distance of *r*_*e*_ = 4.53 Å, and a vibrational constant of $\omega_{e}$ = 49 cm^−1^. We determined a larger potential depth, a similar result as in our former calculations at the same level of theory [37,42], using the same method (multireference configuration interaction), basis sets, core polarization potentials, and effective core potentials. This multireference configuration interaction approach is suitable for the description of higher states, but at the cost of higher uncertainties for the ground state. The dependence of the permanent electric dipole moment (PEDM) of the ground state on the internuclear distance is shown in Fig. 2. The observed behavior is similar to the one reported in Ref. [25]. The absolute value differs with *d*_*e*_ = 1.00 ea_0_ for our calculation and *d*_*e*_ = 0.69 ea_0_ determined in Ref. [25]. RbCa has a larger PEDM than LiCa [37] and RbSr [42], in agreement with the calculations in Ref. [25], where RbCa showed the largest PEDM of several investigated Ak–Ake molecules. Positive electronic dipole moments refer to a net negative charge on the Ca end and a net positive charge on Rb for our selection of the coordinate system. The PEDM is positive at all considered internuclear distances, which indicates an increased probability density for electrons around the Ca atom.

### 4.3 Lowest excited states

**Fig. 3:**
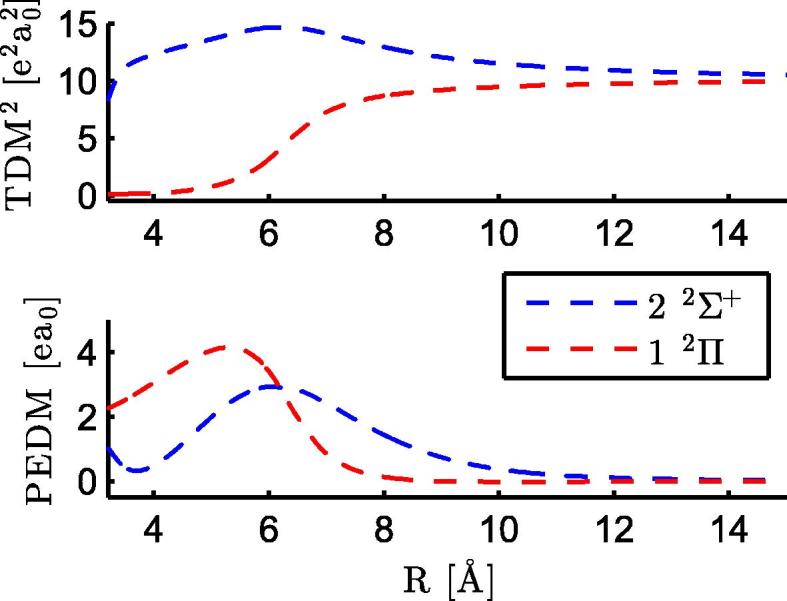
The upper panel of the figure contains the square of the TDMs for a transition from the ground state into the excited states of the diatomic molecule RbCa corresponding to the Rb (5p ^2^P°) + Ca (4s^2^ ^1^S) asymptote. The lower panel presents the PEDMs of the same states.

The two lowest excited states of the RbCa molecule, $2{}^{2}\Sigma^{+}$ and $1{}^{2}\Pi$, correlate to the Rb (5p ^2^P°) + Ca (4s^2^ ^1^S) asymptote. They were only treated theoretically, because the excitation energies (see Table 2) lie too far in the infrared for our laser systems. Fig. 3 shows the corresponding TDMs and PEDMs. The TDMs between the ground state and these excited states are the highest among all transitions in the asymptotic limit (at 30 Å), which is expected from the transitions of the atomic constituents, i.e. Rb (5p ^2^P°) ← Rb (5s ^2^S) [76]. For the $2{}^{2}\Sigma^{+}$ state, the TDM even increases for smaller internuclear separations, resulting in the highest TDM for all treated states. The PEDMs are positive with a single maximum, the $2{}^{2}\Sigma^{+}$ state has its maximum at larger internuclear separation. Both states have similar amplitudes of the PEDMs. The same behavior, at slightly larger internuclear separations, was observed for the two lowest states of RbSr with similar amplitudes, see Fig. 2 in Ref. [42]. This could be interpreted as a contribution of the 5p of Rb to a bonding orbital. For LiCa, the $2{}^{2}\Sigma^{+}$ state shows only a small positive maximum, but the dent, as seen for RbCa in Fig. 3, is more pronounced and results in negative values [37]. It is probably related to the much smaller 2p orbital of Li.

### 4.4 Excitation spectrum from 13 000 to 16 500 cm^−1^

**Fig. 4:**
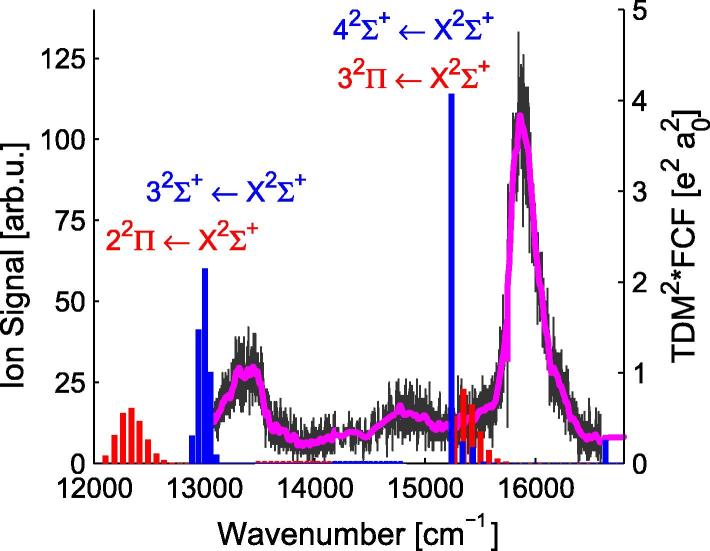
REMPI-TOF excitation spectrum in the range of 13 000–16 500 cm^−1^, the original data points are shown in gray, the magenta line shows the data smoothed by convolution with a Gaussian. The transition probabilities (FCF∗TDM^2^) are shown as vertical sticks, in blue for transitions into ${}^{2}\Sigma^{+}$ states and in red for transitions into ${}^{2}\Pi$ states. (For interpretation of the references to color in this figure legend, the reader is referred to the web version of this article.)

The REMPI-TOF signal in Fig. 4 has been achieved with a two-color two-photon ionization up to ∼14 000 cm^−1^ and with a one-color two-photon ionization above this excitation energy. The threshold energy for a one-color two-photon ionization can only be roughly estimated. The ground state potential well depth of the RbCa molecule was only treated here and in one other publication [25] and can be estimated as 1200 cm^−1^. There are two publications in which the ground and several excited states of the RbCa^+^ cation have been computed. Therein a potential depth of about ∼4100 cm^−1^ and equilibrium separation of ∼8 ${\text{a}}_{0}$ have been reported [50,49], in good agreement with our result (see Table 2). Using these depths, one-color two-photon ionization may become possible around ∼15 400 cm^−1^. Additionally, the effects of the helium environment have to be considered (known to lower the ionization threshold [81,82]). We started the one-color two-photon ionization at 14 000 cm^−1^ in order to detect a possible rise of the signal in the energy range where the ionization threshold was expected. The slight rise in the signal around ∼14 800 cm^−1^ might be an indicator for this, but cannot be assigned with certainty.

**Fig. 5:**
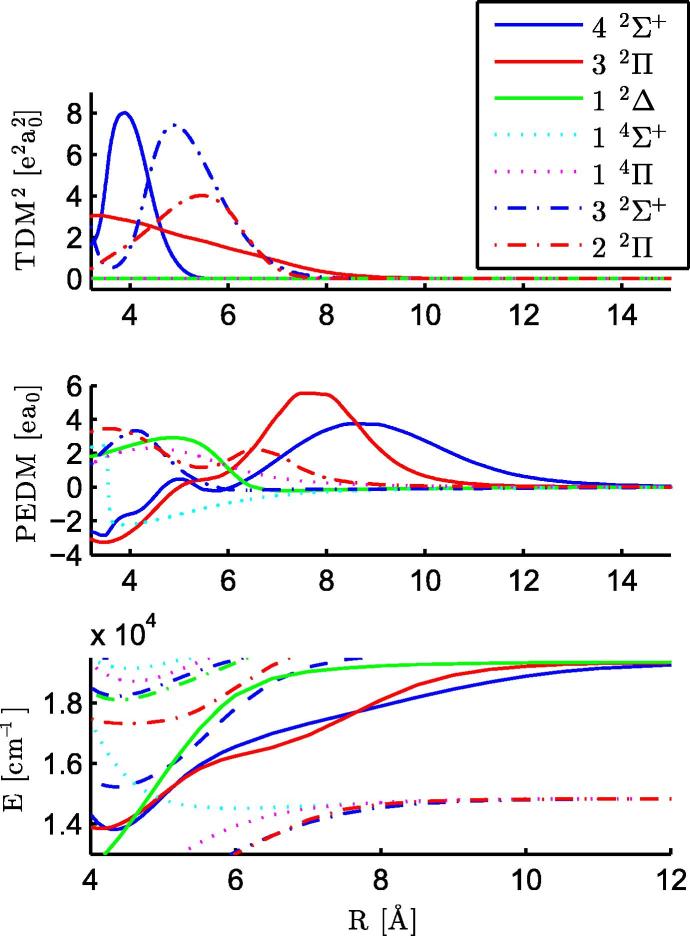
TDMs, PEDMs and PECs for excited states of the RbCa molecule corresponding to the Rb (5s ^2^S) + Ca (4s4p ^3^P°) and Rb (4d ^2^D) + Ca (4s^2^ ^1^S) asymptotes are shown in this figure. These states give rise to the signal in the REMPI-TOF spectrum in Fig. 4.

The small structure at 13 000 cm^−1^ is assigned to the $3{}^{2}\Sigma^{+}$ ← X${}^{2}\Sigma^{+}$ transition on basis of theoretical calculations. The $3{}^{2}\Sigma^{+}$ state is related to the Rb (5s ^2^S) + Ca (4s4p ^3^P°) asymptote. As shown in Fig. 4, the calculations underestimate the position by about ∼200 cm^−1^. This can be explained by the fact that the PEC approaches a value approximately ∼400 cm^−1^ below the Rb (5s ^2^S) + Ca (4s4p ^3^P°) asymptote for large internuclear separations, see Table 1. A comparison with RbSr [37] shows that the $3{}^{2}\Sigma^{+}$ state of RbCa lies ∼600 cm^−1^ higher in energy, which is as expected since the Ca (4s4p ^3^P°) state also lies ∼560 cm^−1^ [76] above the corresponding Sr state. The $2{}^{2}\Pi$ state also has a significant TDM at the equilibrium distance (see Fig. 5), but is more strongly bound than the $3{}^{2}\Sigma^{+}$ state. Therefore this state lies below the experimentally recorded wave length range. The PEDM of the $1{}^{4}\Sigma^{+}$ state becomes negative close to 4 Å, whereas the other three states corresponding to this asymptote show positive PEDMs, see Fig. 5.

The structure around ∼15 900 cm^−1^ in Fig. 4 shows a steep rising edge on the low energy side and a broad shoulder to higher energies. The calculations suggest an assignment of this structure to the molecular states originating from the Rb (4d ^2^D) + Ca (4s^2^ ^1^S) asymptote. According to the calculated transition probabilities in Fig. 4 and the PECs in Fig. 1, the transitions into these two molecular states are overlapped. Hence, the structure at 15 900 cm^−1^ is assigned to the superimposed $3^{2}\Pi /4^{2}\Sigma^{+}\leftarrow X^{2}\Sigma^{+}$ transitions. The $4^{2}\Sigma^{+}$ state has shown vibrational resolution for LiCa [37] as well as for RbSr [45]. There are several reasons for the different structure of the $4^{2}\Sigma^{+}\leftarrow X^{2}\Sigma^{+}$ excitation spectrum of RbCa compared to LiCa and RbSr counterparts. In LiCa and RbSr, the $4^{2}\Sigma^{+}$ state correlates to a ^3^D state asymptote of the alkaline earth, which corresponds to the $6^{2}\Sigma^{+}$ state in RbCa. The $4^{2}\Sigma^{+}$ state of RbCa, however, dissociates into ground state Ca and Rb (4d ^2^D). Ground and excited state potentials look similar for this transition in RbCa with the ($\nu^{\prime }=0-\nu^{\prime \prime }=0$) band having a Franck–Condon factor of about 0.9. The single vertical blue bar in Fig. 4 represents this result and is followed to higher energies by some small structure that stand for the higher $\nu^{\prime }$ levels of $4^{2}\Sigma^{+}$ and the weaker but strongly overlapping $3^{2}\Pi \leftarrow X^{2}\Sigma^{+}$ excitation bands. In LiCa and RbSr the corresponding transitions are well separated.

The spectral range between 14 450 and 16 450 cm^−1^ was additionally investigated with laser induced fluorescence spectroscopy in order to find an emission signal of RbCa. In this wavelength range, both, a Rb_2_ (Rb_2_ ${\left(1\right)}^{1}\Pi_{u}\leftarrow a^{1}\Sigma_{g}^{+}$ [65]) and a Ca_2_ (Ca_2_ $A^{1}\Sigma_{u}^{+}\leftarrow X^{1}\Sigma_{g}^{+}$ [83]) transition can be found. The Rb dimer transition overlaps with a Rb_3_ transition (Rb_3_ $3^{4}E^{\prime }\leftarrow 1^{4}A_{2}^{\prime }$ [65]). However, no emission could be found for RbCa excited around ∼15 900 cm^−1^ in the laser induced fluorescence spectrum. The complete absence of fluorescence can either be explained by a non-radiative relaxation of the RbCa molecules into low lying excited states, from which an emission is beyond our detection limit, or by the transfer of excited RbCa into metastable states. Helium mediated relaxation into the metastable $1^{2}\Delta$ and further to the $1{}^{4}\Pi$ states might be possible. For free molecules, fluorescence emission from $4^{2}\Sigma^{+}$ and $3^{2}\Pi$ can be expected.

### 4.5 Excitation spectrum from 16 500 to 19 500 cm^−1^

**Fig. 6:**
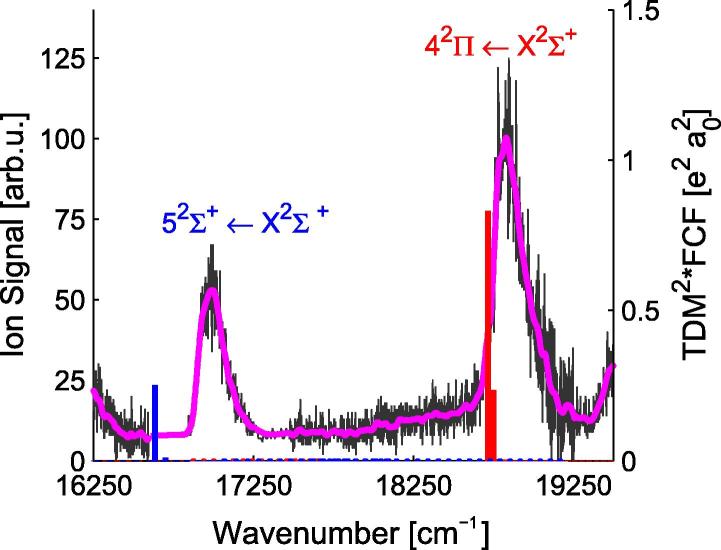
REMPI-TOF excitation spectrum in the range of 16 500–19 500 cm^−1^, the original data points are shown in gray, the magenta line shows the data smoothed by convolution with a Gaussian. The transition probabilities (FCF∗TDM^2^) are shown as vertical sticks, in blue for transitions into ${}^{2}\Sigma^{+}$ states and in red for transitions into ${}^{2}\Pi$ states. (For interpretation of the references to color in this figure legend, the reader is referred to the web version of this article.)

The REMPI-TOF spectrum for transitions in the range of 16 500–19 500 cm^−1^ is shown in Fig. 6. Two peaks at 17 000 cm^−1^ and 18 800 cm^−1^ can be identified, which have been assigned to the molecular $5^{2}\Sigma^{+}\leftarrow X^{2}\Sigma^{+}$ and $4^{2}\Pi \leftarrow X^{2}\Sigma^{+}$ transitions, respectively. The assignment is based on the comparison to calculations, which are shown in form of stick spectra in Fig. 6.

**Fig. 7:**
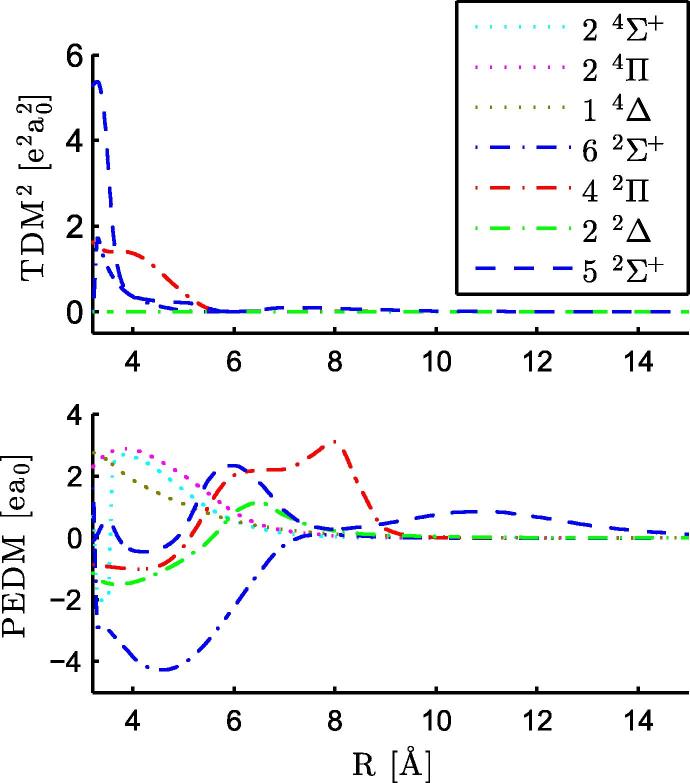
PEDMs and TDMs for excited states of the RbCa molecule related to the Rb (6s ^2^S) + Ca (4s^2^ ^1^S) and Rb (5s ^2^S) + Ca (3d4s ^3^D) asymptotes. The $4^{2}\Pi$ and $5^{2}\Sigma^{+}$ states give rise to a signal in the REMPI-TOF spectrum in Fig. 6.

Both states are underestimated by the calculations (∼300 cm^−1^ for the $5^{2}\Sigma^{+}$ state and ∼40 cm^−1^ for the $4^{2}\Pi$ state). For the $5^{2}\Sigma^{+}$ state, this can be explained by an underestimation of the asymptotic value by the theoretical calculations as can be seen in Table 1, which also affects the PEC at smaller internuclear separations. In contrast, the asymptotic value of the $4^{2}\Pi$ state is overestimated by a few hundred cm^−1^, but there is an avoided crossing between the $4^{2}\Pi$ and $5{}^{2}\Pi$ states at 9 Å, see the PECs in Fig. 9. This avoided crossing results in a strong change of the PEDM at the same distance, as can be seen in Fig. 7. The $5^{2}\Sigma^{+}$ state has a negative PEDM and corresponds to the Rb (6s ^2^S)+ Ca (4s^2^ ^1^S) asymptote, this negative value was also obtained for the same state in RbSr. The TDMs in the upper part in Fig. 7 are small beyond an internuclear separation of 5 Å, but become large at the equilibrium distance of the ground state. For both transitions, the Franck–Condon factors favor ($\nu^{\prime }=0-\nu^{\prime \prime }=0$) transitions. Again, no fluorescence was detected after excitation of the observed RbCa bands.

### 4.6 Excitation spectrum from 19 500 to 23 000 cm^−1^

**Fig. 8:**
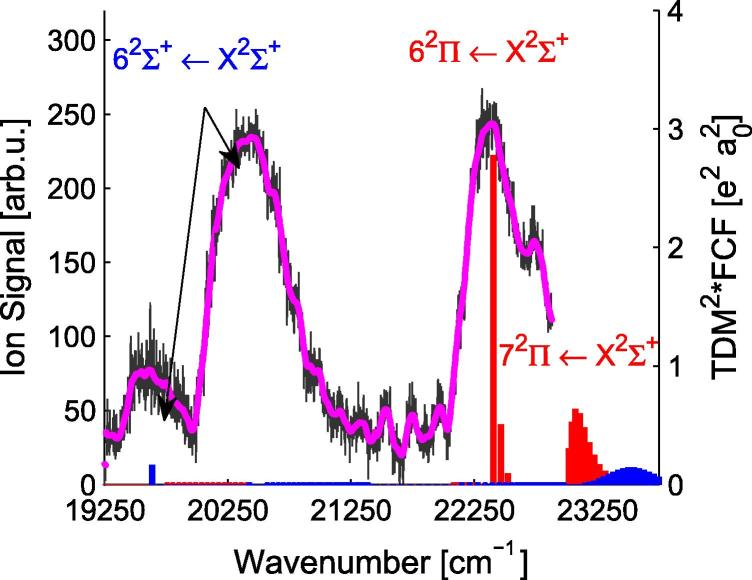
REMPI-TOF excitation spectrum in the range of 19 500–23 000 cm^−1^, the original data points are shown in gray, the magenta line shows the data smoothed by convolution with a Gaussian. Between the two strong transitions around 20 400 cm^−1^ and 22 400 cm^−1^ a resolved structure can be seen. The transition probabilities (FCF∗TDM^2^) are shown as vertical sticks, in blue for transitions into ${}^{2}\Sigma^{+}$ states and in red for transitions into ${}^{2}\Pi$ states. (For interpretation of the references to color in this figure legend, the reader is referred to the web version of this article.)

**Fig. 9:**
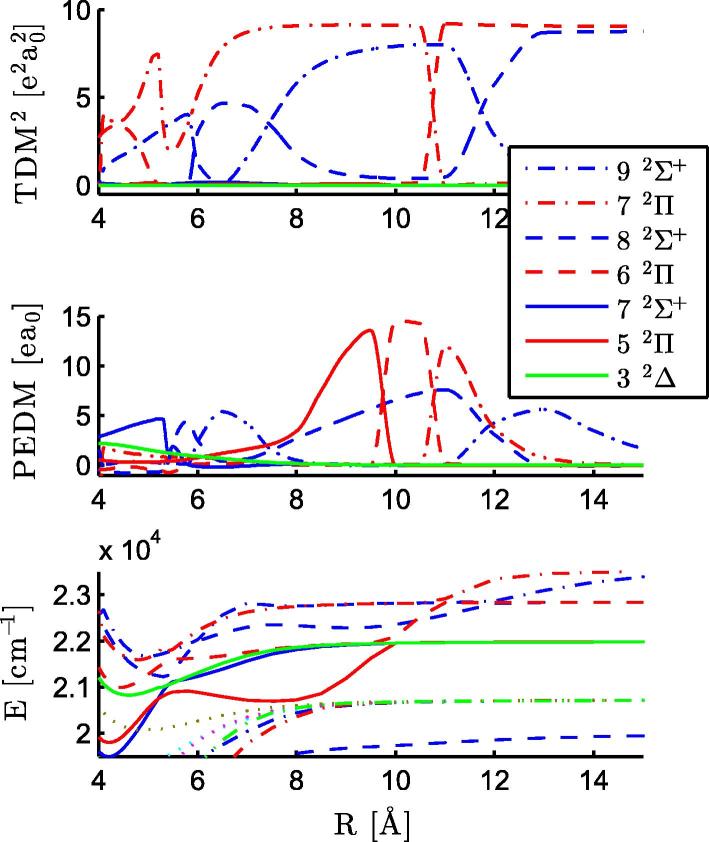
TDMs, PEDMs, and PECs for excited states of RbCa with the Rb (5s ^2^S) + Ca (3d4s ^1^D), Rb (5s ^2^S) + Ca (4s4p ^1^P°), and Rb (6p ^2^P°) + Ca (4s^2^ ^1^S) asymptotes are displayed. The abrupt changes are related to avoided crossings, where the molecular states change their character.

Fig. 8 shows the excitation spectrum of RbCa above 19 500 cm^−1^ as recorded with REMPI-TOF spectroscopy. Several distinct structures can be identified. Between the two broad structures at 20 400 cm^−1^ and 22 400 cm^−1^ several resolved lines appear. The peaks are Gauss-shaped and have a spacing of ∼200(10) cm^−1^. The series can be followed from the maximum of the peak of the lower lying structure (20 400 cm^−1^) to the onset of the transition at higher energies (22 100 cm^−1^). These structures have been reproduced in several experiments, but could not be separated better from the energetically higher or lower-lying transitions. The peaks only occur for the mass window of the RbCa isotopologues and a contribution of dimers or triatomic molecules (Rb_2_Ca or Ca_2_Rb) can be excluded. However, neither the calculations nor the comparison to RbSr indicate a PEC for such high excitation energies, bound deeply enough to harbor vibrational lines with a spacing of ∼200(10) cm^−1^ (see Table 2). The $7^{2}\Sigma^{+}$ and $5^{2}\Pi$ states lie in this range, but their vibrational spacing is significantly smaller and they have negligible TDMs, see Fig. 9. This structure might be related to the first excited state of the cation, $1{}^{3}\Sigma^{+}$ [49–51]. It lies at about 43 000 cm^−1^, an energy that can be supplied with two photons in this energy range.

The structure at 20 400 cm^−1^ is tentatively assigned to the $6^{2}\Sigma^{+}\leftarrow X^{2}\Sigma^{+}$ transition, since the peak around 19 600 cm^−1^ has been found not to originate from a RbCa molecular transition, *vide infra*. Theoretical calculations suggest the assignment of the structure at 22 400 cm^−1^ to the $6^{2}\Pi \leftarrow X^{2}\Sigma^{+}$ transition and the shoulder at higher energies (22 700 cm^−1^) to the $7^{2}\Pi \leftarrow X^{2}\Sigma^{+}$ transition. The TDMs for transitions into ${}^{2}\Sigma^{+}$ states above $6^{2}\Sigma^{+}$ are negligible at 4.4 Å, see Fig. 9.

The strong interactions of the energetically highest states with each other are obvious in Fig. 9. The TDMs and the PEDMs change their values, indicating avoided crossings, which are observable in Figs. 1 and 9. The two states corresponding to the Rb (6p ^2^P°) + Ca (4s^2^ ^1^S) asymptote ($9^{2}\Sigma^{+}\text{,}7^{2}\Pi$) show avoided crossings at 11 and 12 Å and corresponding changes in the PEDMs and TDMs. These two states are influenced by higher states for small internuclear separations.

### 4.7 Structure at 19 600 cm^−1^

**Fig. 10:**
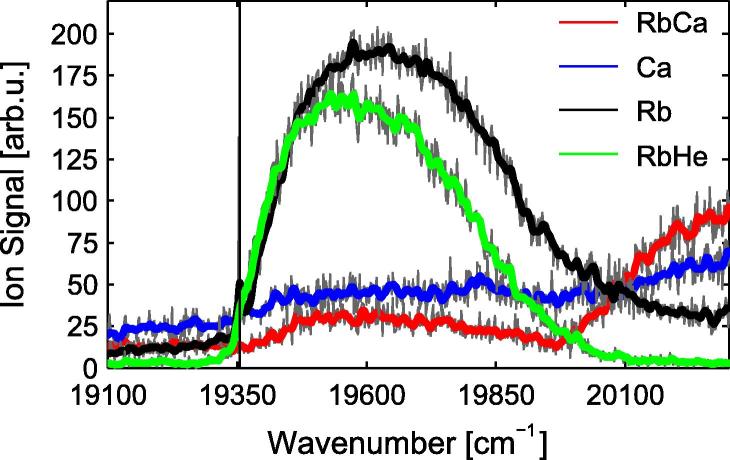
Close-up of the transition around 19 600 cm^−1^ in the RbCa ion yield. The ion yields for ^40^Ca (blue), ^85^Rb+^87^Rb (black), ^85^RbHe+^87^RbHe (green) and ^85^Rb^40^Ca+^87^Rb^40^Ca (red) are shown, the vertical line indicates the free atom Rb (4d ^2^D) ← Rb (5s ^2^S) transition. It can be seen that the RbCa signal follows the Rb and Rb-He signal. The peak in the RbCa ion yield is not correlated to a RbCa transition, as explained in the text. (For interpretation of the references to color in this figure legend, the reader is referred to the web version of this article.)

Fig. 8 reveals the presence of a small peak slightly below the $6^{2}\Sigma^{+}\leftarrow X^{2}\Sigma^{+}$ transition with a maximum around 19 600 cm^−1^. A comparison of different ion traces recorded in the REMPI experiment suggests that this structure is not related to a RbCa transition. The REMPI spectra for different ions are presented in Fig. 10, the ion yield originating from RbCa is shown in red, Ca in blue, Rb in black and RbHe in green. The atomic Rb (4d ^2^D) ← Rb (5s ^2^S) transition is indicated by the vertical black line. It is remarkable that the main feature in Fig. 10 can be unambiguously identified in the Rb, RbHe and the RbCa ion signal. Also, the Ca signal seems to weakly follow the other signals. The rising edge of the transition coincides exactly with the free atom transition in Rb, followed by a blue shifted maximum and an extended blue wing. This is the typical form of Rb-He_*N*_ transitions in the lower energy region [84–87]. Following the pseudo-diatomic molecule notation for alkali doped helium droplets [88,84], this transition corresponds to the merged 4^2^D (${}^{2}\Pi$) ← 5^2^S_1/2_ (${}^{2}\Sigma_{1/2}$) and 4^2^D (${}^{2}\Sigma$) ← 5^2^S_1/2_ (${}^{2}\Sigma_{1/2}$) transitions in Rb-He_*N*_. These transitions have been previously observed [87,89] and are in excellent agreement with the REMPI spectra in Fig. 10, consequently we assign the Rb and RbHe ion signal to these transitions.

The observation of this transition in the Rb and RbHe signal is not surprising. Due to the statistical nature of the doping process, monomers are always present at doping conditions optimized for RbCa. However, it is remarkable that the RbCa and, very weakly, the Ca signal follow the Rb-He_*N*_ transition. This can only be explained if it is assumed that a fraction of Rb and Ca atoms are initially found separated from each other on the helium droplet. The laser excitation and subsequent ionization of the Rb atom changes the interaction with the helium environment. (Neutral Rb is heliophobic, but the Rb ion is heliophilic [90].) This initiates a dynamic process in which the Rb ion (or excited atom) finds a Ca atom and forms RbCa, which explains the RbCa signal. Subsequent fragmentation of a fraction of the formed molecular ions may lead to a weak signal in the Ca mass window. Similar processes have been observed for Na and K doped He droplets in Ref. [63].

## 5 Conclusion

In this manuscript we applied a combined theoretical and experimental approach in the analysis of RbCa. *Ab initio* calculations were performed for 24 electronic states of RbCa and their energies and permanent electric dipole moments were determined. The combination of the transition dipole moments at equilibrium position of the ground state and the Franck–Condon factors allowed to calculate a theoretical spectrum, which was subsequently compared to experimental measurements. In the experiment, the RbCa molecule was synthesized on helium nanodroplets by a sequential pick up scheme and then analyzed by resonance enhanced multiphoton ionization time-of-flight spectroscopy. With the aid of the theoretical results seven spectroscopic bands were assigned to excited states of RbCa, and one feature to an excitation of the Rb atom.

RbCa is the third Ak–Ake molecule in a series of combined He nanodroplet and *ab initio* investigations. In contrast to LiCa and RbSr, the relaxation mechanisms seem to be more complicated and no fluorescence was observed. These mechanisms probably relate to the He environment.

Besides the current interest of the cold molecule community, there is the need for a deeper understanding of the bonding mechanisms in this group of molecules. The group of alkaline earth monohalides was well described in terms of ionic bonding models either based on mutual polarization of the atomic constituents [91,92] or on a ligand field approach [93]. The ligand field theory was also successful in the development of a model for the electronic structure of alkaline earth oxides, first for CaO [94,95], and later applied to SrO [96]. A large amount of experimental data was available for these molecules at that time, whereas we are just at the beginning of data collection for alkali–alkaline earth diatomic molecules. While we are currently working on an analysis of the common properties of these molecules, we can summarize already a few peculiarities found in this study of RbCa. If we concentrate on the ${}^{2}\Sigma^{+}$ and ${}^{2}\Pi$ states, the ground state exhibits the by far smallest bonding energy. Among the excited states, those correlating to excited states of Ca, show a smaller well depth than those correlating to excited Rb states. Up to $3^{2}\Sigma^{+}$ and $2^{2}\Pi$, the permanent electric dipole moment at *r*_*e*_ = 4.37 Å (ground state equilibrium internuclear distance) are positive irrespective of their asymptote. From $4^{2}\Sigma^{+}$ and $3^{2}\Pi$ up in energy, the dipole moment of most states is negative. Our studies have focused on combinations relevant for ultracold molecular physics. The helium nanodroplet isolation method shows promise for further investigations of diatomic molecules. Future applications of this approach may envisage the production of more exotic species, which are not easily accessible by conventional methods.

## Figures and Tables

**Table 1:** This table compares atomic energy levels as found in the NIST-database to calculated asymptotic excitation energies (30 Å). The values are given in cm^−1^.

State	NIST [76]	This work
Rb (5p ^2^P°)	12 737	12 516
Ca (4s4p ^3^P°)	15 263	14 849
Rb (4d ^2^D)	19 355	19 367
Rb (6s ^2^S)	20 133	19 962
Ca (3d4s ^3^D)	20 357	20 721
Ca (3d4s ^1^D)	21 850	21 987
Ca (4s4p ^1^P°)	23 652	22 843
Rb (6p ^2^P°)	23 767	23 525


**Table 2:** Parameters for the calculated PECs of the RbCa diatomic molecule are shown in the table. The potential depth (*D*_*e*_), the vibrational constant ($\omega_{e}$) and the excitation energy (*T*_0_) are given in cm^−1^. The values of the internuclear distances (*r*_*e*_) are given in Å. *T*_0_ (exp.) refers to the position of the rising edges of the experimentally observed transitions. The corresponding asymptote for each state is denoted in the last column.

State	*r*_*e*_	$\omega_{e}$	*D*_*e*_	*T*_0_ (calc.)	*T*_0_ (exp.)	Asymptote
X ${}^{2}\Sigma^{+}$	4.37	58	1406			Rb (5s ^2^S) + Ca (4s^2^ ^1^S)
2 ${}^{2}\Sigma^{+}$	4.34	78	5446	8487		Rb (5p ^2^P°) + Ca (4s^2^ ^1^S)
1 ${}^{2}\Pi$	3.75	108	9175	4772		Rb (5p ^2^P°) + Ca (4s^2^ ^1^S)
3 ${}^{2}\Sigma^{+}$	4.02	62	3367	12 891	13 100	Rb (5s ^2^S) + Ca (4s4p ^3^P°)
2 ${}^{2}\Pi$	4.02	79	4156	12 110		Rb (5s ^2^S) + Ca (4s4p ^3^P°)
1 ${}^{4}\Sigma^{+}$	5.98	19	336	15 901		Rb (5s ^2^S) + Ca (4s4p ^3^P°)
1 ${}^{4}\Pi$	4.13	77	3220	13 045		Rb (5s ^2^S) + Ca (4s4p ^3^P°)
4 ${}^{2}\Sigma^{+}$	4.32	96	5551	15 241	15 700	Rb (4d ^2^D) + Ca (4s^2^ ^1^S)
3 ${}^{2}\Pi$	4.14	79	5523	15 261	15 700	Rb (4d ^2^D) + Ca (4s^2^ ^1^S)
1 ${}^{2}\Delta$	3.74	100	7014	13 781		Rb (4d ^2^D) + Ca (4s^2^ ^1^S)
5 ${}^{2}\Sigma^{+}$	4.34	68	4739	16 635	16 900	Rb (6s ^2^S)+ Ca (4s^2^ ^1^S)
6 ${}^{2}\Sigma^{+}$	4.36	63	2492	19 638	20 100	Rb (5s ^2^S) + Ca (3d4s ^3^D)
4 ${}^{2}\Pi$	4.48	35	3405	18 712	18 750	Rb (5s ^2^S) + Ca (3d4s ^3^D)
2 ${}^{2}\Delta$	4.39	58	2621	19 507		Rb (5s ^2^S) + Ca (3d4s ^3^D)
2 ${}^{4}\Sigma^{+}$	4.52	47	1574	20 549		Rb (5s ^2^S) + Ca (3d4s ^3^D)
2 ${}^{4}\Pi$	4.59	47	2001	20 121		Rb (5s ^2^S) + Ca (3d4s ^3^D)
1 ${}^{4}\Delta$	4.92	28	653	21 460		Rb (5s ^2^S) + Ca (3d4s ^3^D)
7 ${}^{2}\Sigma^{+}$	4.20	77	2498	20 906		Rb (5s ^2^S) + Ca (3d4s ^1^D)
5 ${}^{2}\Pi$	4.21	74	2192	21 210		Rb (5s ^2^S) + Ca (3d4s ^1^D)
3 ${}^{2}\Delta$	4.59	40	1158	22 227		Rb (5s ^2^S) + Ca (3d4s ^1^D)
8 ${}^{2}\Sigma^{+}$	5.19	59	1618	22 632		Rb (5s ^2^S) + Ca (4s4p ^1^P°)
6 ${}^{2}\Pi$	4.47	59	1843	22 406	22 100	Rb (5s ^2^S) + Ca (4s4p ^1^P°)
9 ${}^{2}\Sigma^{+}$	5.00	42	1855	23 069		Rb (6p ^2^P°) + Ca (4s^2^ ^1^S)
7 ${}^{2}\Pi$	4.80	49	1933	22 994	22 700	Rb (6p ^2^P°) + Ca (4s^2^ ^1^S)
RbCa^+^	4.24	75	3925	30 972		Rb^+^ (^1^S) + Ca (4s^2^ ^1^S)


## Footnotes

2 The PECs, TDMs and PEDMs are available as Supplementary data.
